# Effect of Fear, Concern, and of Risk Factors for Complicated Covid-19 on Self-Care in People in Pre-elderly and Elderly Stages*

**DOI:** 10.17533/udea.iee.v40n3e10

**Published:** 2023-02-13

**Authors:** Josué Arturo Medina-Fernández, Isaí Arturo Medina-Fernández, Nissa Yaing Torres-Soto, Luis Carlos Cortez-González, Fernanda Guadalupe Rascón-Arriaga, Diana Berenice Cortes-Montelongo

**Affiliations:** 1 Master’s in Nursing, Full-time research professor, Division of Health Sciences, Universidad Autónoma del Estado de Quintana Roo, Sede Chetumal. E-mail: josuemedinafernandez@outlook.es Universidad de Quintana Roo Division of Health Sciences Universidad Autónoma del Estado de Quintana Roo Chetumal Mexico josuemedinafernandez@outlook.es; 2 Master’s in Nursing, Full-time research professor, Faculty of Nursing “Dr. Santiago Valdés Galindo”, Universidad Autónoma de Coahuila, Sede Saltillo. E-mail: isai-medina@uadec.edu.mx Universidad Autónoma de Coahuila Faculty of Nursing Universidad Autónoma de Coahuila Saltillo Mexico medina@uadec.edu.mx; 3 PhD in Social Sciences, Full-time research professor, Division of Health Sciences, Universidad Autónoma del Estado de Quintana Roo, Sede Chetumal. E-mail: nissa.torres@uqroo.edu.mx Universidad de Quintana Roo Division of Health Sciences Universidad Autónoma del Estado de Quintana Roo Chetumal Mexico nissa.torres@uqroo.edu.mx; 4 PhD in Nursing Sciences, Full-time research professor, Faculty of Nursing “Dr. Santiago Valdés Galindo”, Universidad Autónoma de Coahuila, Sede Saltillo. E-mail: lucortezg@uadec.edu.mx Universidad Autónoma de Coahuila Faculty of Nursing Universidad Autónoma de Coahuila Saltillo Mexico lucortezg@uadec.edu.mx; 5 PhD in Social Sciences, Professor and Researcher, Universidad Kino. E-mail: fernanda_rascon@unikino.edu.mx Universidad Kino Universidad Kino Mexico fernanda_rascon@unikino.edu.mx; 6 PhD in Nursing Sciences, Full-time research professor, Faculty of Nursing “Dr. Santiago Valdés Galindo”, Universidad Autónoma de Coahuila, Sede Saltillo. E-mail: dicortesm@uadec.edu.mx Universidad Autónoma de Coahuila Faculty of Nursing Universidad Autónoma de Coahuila Saltillo Mexico dicortesm@uadec.edu.mx

**Keywords:** fear, expression of concern, self-care, adult, aged, COVID-19., miedo, expresión de preocupación, autocuidado, adulto, anciano, COVID-19., medo, expressão de preocupação, autocuidado, adulto, idoso, COVID-19.

## Abstract

**Objective.:**

The work sought to determine the effect of the risk factors, fear, and concern on self-care regarding COVID-19 in people in pre-elderly and elderly stages.

**Methods.:**

Correlational-predictive study, gathered through convenience sampling. The study applied the scale of fear of COVID-19 (Huarcaya *et al*.), the scale of concern about COVID-19 (Ruíz *et al*.,), and the scale of self-care during the COVID-19 confinement (Martínez *et al.,*). Descriptive and inferential statistics were applied as the mediation model based on regression.

**Results.:**

The study had the participation of 333 people, with the majority being women (73.9%). Correlation was found between self-care with the scores from the scale of fear (r = -0.133, *p* <0.05) and of concern (r = -0.141, *p*<0.05) regarding COVID-19. The direct effect of the model was c’ = 0.16, [95% BCa CI = -0.28, -0.09]. The standardized value for the indirect effect was estimated as c = -0.14, [ Ca CI= -0.23,-0.09], which shows existence of a 14.0% effect of the mediating variable on self-care conducts in the prediction model.

**Conclusion.:**

A direct effect exists of risk factors for COVID-19 complication on self-care, mediated by concern and fear, besides explaining by 14% the self-care conducts for COVID-19. Recommendation is made to address other emotional variables to consider if these increase the prediction.

## Resumen

**Objetivo.:**

Determinar el efecto de los factores de riesgo, temor y preocupación sobre el autocuidado ante la COVID-19 en personas en etapa de prevejez y vejez.

**Métodos.:**

Estudio correlacional-predictivo, recolectado mediante un muestreo a conveniencia. Se aplicó la escala de temor ante el COVID-19 (Huarcaya *et al*.), la escala de preocupación ante el COVID-19 (Ruíz *et al*.) y la escala de autocuidado durante el confinamiento de COVID-19 (Martínez *et al.*). Se aplicó estadística descriptiva e inferencial como el modelo de mediación basado en regresión.

**Resultados.:**

Participaron 333 personas, en su mayoría mujeres, 73.9%. Se halló correlación entre el autocuidado con los puntajes de la escala de temor (r=-0.133, *p*<0.05) y de preocupación (r=-0.141, *p*<0.05) ante la COVID-19. El efecto directo del modelo fue de c’=0.16, [Ca CI =-0.28,-0.09]. Por su parte, el valor estandarizado para el efecto indirecto se estimó en c=-0.14, [Ca CI= -0.23,-0.09], lo que muestra la existencia de un efecto de 14.0% de la variable mediadora sobre las conductas de autocuidado en el modelo predictor.

**Conclusión.:**

Existe un efecto directo de los factores de riesgo para complicación de la COVID-19 sobre el autocuidado, mediado por la preocupación y el temor, aunado a que explica en un 14% la conducta de autocuidado para la COVID-19. Se recomienda abordar otras variables emocionales para considerar si estas aumentan la predicción.

## Resumo

**Objetivo.:**

Determinar o efeito dos fatores de risco, medo e preocupação no autocuidado diante da COVID-19 em pessoas na fase de velhice e velhice.

**Métodos.:**

Estudo preditivo-correlacional, coletado por amostragem de conveniência. A escala de medo do COVID-19 foi aplicada (Huarcaya *et al.*), a escala de preocupação COVID-19 (Ruíz *et al*.) e a escala de autocuidado durante o confinamento COVID-19 (Martínez *et al.*). Estatísticas descritivas e inferenciais foram aplicadas como modelo de mediação baseado em regressão.

**Resultados.:**

Participaram 333 pessoas, sendo a maioria mulheres 73,9%. Foi encontrada correlação entre o autocuidado e os escores da escala de medo (r=-0.133, *p*<0.05) e preocupação (r=-0.141, *p*<0.05) diante da COVID-19. O efeito direto do modelo foi c'=0,16, [Ca CI =-0.28,-0.09>. Por sua vez, o valor padronizado para o efeito indireto foi estimado em c=-0.14, [Ca CI= -0.23,-0.09> o que mostra a existência de um efeito de 14.0% da variável mediadora sobre o self -comportamentos de cuidado no modelo preditor.

**Conclusão.:**

Há efeito direto dos fatores de risco para complicações da COVID-19 no autocuidado, mediado pela preocupação e medo, além de explicar 14% do comportamento de autocuidado para a COVID-19. Recomenda-se abordar outras variáveis emocionais para considerar se elas aumentam a previsão.

## Introduction

On 11 March 2020, the World Health Organization declared a global pandemic caused by Severe Acute Respiratory Syndrome Coronavirus*2*(SARS-CoV-2), designated as COVID-19. By early February 2022, over 5.15-million accumulated cases of COVID-19 had been registered in Mexico. The COVID-19 pandemic has affected all people; however, it has not been the same for everyone, given that big differences exist in health risks and self-care of the people.(1) Thus, during the course of the epidemic, the disease, as well as the health problems, fear, concern, which take place and are faced, negotiations in interpersonal relations have evolved, which is why the confinement and social distancing have been among the principal strategies generated by international organizations to curtail the advance of COVID 19; however, the emotional and mental health of the people has been seriously affected, more so in the most-vulnerable population, like those in the pre-elderly stage (between 45 and 59 years of age) and elderly stage (over 60 years of age).(2) Many daily life activities have changed due to the pandemic and the confinement, among these, the perceptions, relationships, their own and group behaviors.(3)

Moreover, by nature, humans are sociable beings, relationships are a fundamental part in the development of people and respond to our actions throughout history; instead, within the context of the pandemic unusual measures had to be taken to protect the most vulnerable population, that is how social distancing has led us to diminished contact and relationships among people. Among the principal psychological effects during the pandemic there are fear and concern in relation to COVID-19 and regarding the possible contagion of individuals and their families. For this reason, fear is considered a fundamental emotion for human survival, caused by a real threat that triggers distinctive reactions of fear or alarm, through physical and psychological responses and change.(4) The intensity of the responses of fear and anxiety will be mediated by biological and environmental factors, such as the presence of a chronic disease, overweight, being elderly, lack of knowledge about the disease, the news, personal experience, among others, which is why individual differences in expressing these emotions can range from a response of fear and anxiety, to elevated and even irreversible emotional states, experienced as excessive fear and anxiety.(5)

From the studies reviewed in the literature, negative psychological effects were observed, including symptoms of post-traumatic stress, confusion, and anger.(5) Stressing factors were due to the presence of spending more time in isolation, risk factors for COVID-19 complication, fear of infection, frustration, boredom, inadequate information, economic loss, and stigma about the disease.(6) Some studies even suggest lasting effects, where greater negative effects have been observed in women, in those living in urban areas and having antecedents of diagnosed anxiety.(7,8)

Another emerging theme is concern, given that during the confinement period, concern was related principally with the risk of their own contagion and of their relatives, the economic situation and health situation caused by other diseases.(9) Uncertainty about the treatment, symptoms and duration of the disease can result in diverse degrees of concern among the population and provoke changes in their attitudes and behaviors.(10)

The pandemic threatens different spheres of our lives; it is how fear and concerns can trigger changes in health self-care by people because they can neglect necessary actions to preserve health and those to prevent contagion of COVID 19.(6) During this time of pandemic, self-care is considered a coherent part in preventive treatment against COVID 19, given that it is justified if the person identifies those altered behavioral factors, prevention measures will increase and contagion cases could diminish, being applicable to the various variants that are identified, which is why the perspective of the people helps to be aware of their own health by improving information and skills to carry out the necessary care actions.(11) Likewise, this research is not only important for people undergoing an aging process during the COVID-19 pandemic, but, also as nursing staff, it allows considering those behavioral factors to work on them, coupled with the fact that it is important due to the social relevance and because of the humane care of vulnerable individuals, protecting them from incompetent or unsafe health practices, supporting the person to avoid disproportionate risks of contagion and avoid complications or risk of death.(12,13) Due to the foregoing, the proposal is to determine the influence of fear and concern about self-care regarding COVID-19 in people in pre-elderly and elderly stage.

## Methods

This was a correlational-predictive study. A non-probabilistic convenience sampling was conducted, elaborated with the G Power program, calculating a sample size of 134 participants in pre-elderly and elderly stages, considering 0.05 probability of committing type-1 error, power of 90% (1-β=0.9) and size effect of 0.10. With the aforementioned, a sample of 333 people was obtained in pre-elderly and elderly stages, carried out through a convenience sampling based on the network, this was shared through the internet and other social networks (WhatsApp, Facebook, Instagram, Twitter, etc.), considering the self-applicable collection, or another case, a videoconference was held to apply the surveys. Among the inclusion criteria was being 45 years old or older, having a digital device to fill out the instruments or access to videocalls, and having a social network. 

The characterization and measurement instruments were shared through social networks using Google forms; permission was obtained from the authors to use the instruments. The average time to answer the instruments was 20 min. The study was approved by the ethics and research committee of the Faculty of Nursing at Universidad Autónoma de Coahuila and the electronic informed consent was applied.

Among the measurement instruments, a data card was applied where participant personal data was considered, as well as the presence of COVID-19 in the family, having been diagnosed with the disease, and the number of risk factors for complication regarding this disease, such as suffering from hypertension, diabetes, tobacco use, chronic obstructive pulmonary disease, heart disease, immunosuppression, chronic kidney disease, overweight or obesity.

To measure fear of COVID-19, the scale by the same name was applied; it has seven items, with one-dimensional behavior in construct validity, which reported factor loadings from 0.66 onwards and an item-total correlation > 0.4. In its original version, it reports a Cronbach’s alpha coefficient of 0.82. It has a five-point Likert-type response scale, from never to always. The scale indicates that a higher score means greater fear of COVID-19.(14) To measure concern, the study applied the scale concern for COVID-19, which has seven items, is one-dimensional, with an item correlation > 0.390 and factor loadings > 0.90, and reports Cronbach’s alpha of 0.86. The scale has four-point Likert-type questions and interprets that a higher score indicates greater concern regarding COVID-19.(15) For the variable of self-care regarding COVID-19, the study applied the scale detection of self-care activities during the COVID-19 confinement (SSAS-14) in its Spanish version, comprised of 14 items with a Likert-type scale ranging from 1 to 7, with a Cronbach's alpha of 0.80 and it is interpreted that the higher the score, the greater the self-care activities during the COVID-19 confinement.(16)

The statistical analysis used the SPSS program version 25, which applied descriptive statistics by using means, standard deviation, frequencies and percentages. Correlations were explored among continuous variables from Spearman’s statistic, with prior compliance of normality requirements. 

**Figure 1:**
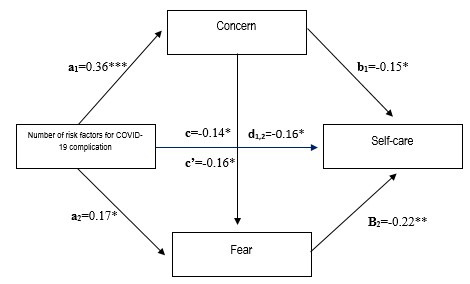
Mediation model among the COVID-19 risk factors and its effect on self-care, mediated by concern and fear (**c**; indirect effect = 0.14, p <0.05 CI ; **c’**, direct effect = 0.16, p<0.05 CI ). Indirect effect: concern, fear, and self-care (d_1,2_=0.16). Note: *p<0.05, **p<0.01, ***p<0.001

To test the mediation model based on regression, the Macro Process was used for the SPSS statistical program(12) to examine the direct and indirect effects of the COVID-19 risk factors on self-care mediated by concern and fear (Figure 1 for the model). A multiple mediation analysis was performed with two mediating variables forming a causal chain. The PROCESS program is a macro (computational) tool that simplifies implementation of mediation, moderation, and analysis of conditional processes using manifest variables. Each estimation by PROCESS requires at least two regression equations and uses ordinary least-squares regression to calculate the parameters of each equation.(17,18)

## Results

The study was made up of 333 participants of which 73.9% (246) were women and 26.1% (87) men, with mean age of 53.91 ±7.43 years; 63.4% (211) were married, followed by those who were divorced 15.6% (52), single 9.3% (31), and common-law 11.7% (39). With respect to COVID-19, 10.5% (35) contracted the disease and 49.8% (166) reported having had a relative with COVID-19, with a mean of 1.44 ±2.14 relatives infected, with minimum and maximum values of 1 to 10 relatives. Regarding the risk factors for complications during the disease, 62.2% reported having at least one risk factor, with a mean of 1.03 ±1.14 and minimum and maximum values of 3 and 9 factors, respectively, observing mostly overweight/obesity, diabetes and hypertension. 

Table 1 shows the sample’s descriptive data; also, adults in elderly stage had greater fear, concern, and self-care regarding COVID-19, observing significant difference in self-care (*p* <0.05). In addition, the group comprised by whether they had endured COVID-19 has greater fear, concern, and self-care, but did not obtain significant difference with the group that had not endured it (*p* >0.05).

**Table 1:** Descriptive statistics of the study variables

Scale	Total		Pre-elderly			Elderly
	M	SD	M	SD	M	SD
Fear of COVID-19	16.73	5.99	16.49	6.09	17.51	5.63
Concern regarding COVID-19	11.69	4.09	11.49	4.03	12.35	4.22
Self-care regarding COVID-19	62.32	12.90	61.49	13.10	65.10	11.53

Note: M = mean; SD = standard deviation.

Table 2 shows significant correlation among the study variables, among them observing that, the older the participant, the greater the number of risk factors for complication of COVID-19 and greater self-care. Likewise, it was found that the greater number of relatives with COVID-19 diagnosis, the greater the fear. Moreover, it was observed that a greater number of risk factors for COVID-19 complication meant greater fear, concern, and self-care. Lastly, it was found that greater self-care indicated less fear and concern regarding COVID-19.

**Table 2:** Correlation of the interpersonal and emotional variables in the participants

	1	2	3	4	5
1. Age	1				
2. Number of relatives with COVID-19 diagnosis	-0.019	1			
3. Number of pathologies as risk factors for COVID-19 complication	0.162**	0.151**	1		
4. Fear of COVID-19	-0.019	0.199**	0.233**	1	
5. Concern regarding COVID-19	-0.045	0.183**	0.276**	0.819**	1
6. Self-care regarding COVID-19	0.54**	-0.043	-0.129*	-0.133*	-0.141**

Note: * =p<0.05, ** = p<0.001

The multiple mediation analysis was tested using a hierarchical regression analysis, as shown in the previous Figure 1. The normalized regression coefficient among the COVID-19 risk factors has a positive and significant effect on concern (a_1_ = 0.36, *t* = -3.4467, *p* <0.001), which - in turn - produces a negative effect on the self-care conducts (b_1_=-0.15, *t*=-3.5679, p <0.01). Similarly, the COVID-19 risk factors affect positively and significantly the presence of fear (a_2_ = 0.17, *t* = -3.6523, *p* <0.05) with r^2^ of 0.2129, indicating that the model explains 21.29% of the variance in self-care. In turn, fear produced a negative and significant effect on self-care (b_2_ = -0.22, *t* = -3.4328, *p* <0.001) with r^2^ of 0.2246, explaining 22.46% of the variance in self-care. Finally, the indirect effect between worry about fear and self-care resulted statistically significant (d_1,2_ = 0.16, p <0.05, CI [-0.29, -0.03]).

The model’s direct effect resulted from *c’* = 0.16, 95% BCa CI [-0.28,-0.09], which indicates existence of an effect of the COVID-19 risk factors on self-care, mediated by concern and fear. In turn, the standardized value for the indirect effect (*c*=-0.14, 95% BCa, CI [-0.23,-0.09]) shows the existence of a 14.0% effect of the mediating variable on the self-care conducts in the prediction model. Although it is an effect considered small, we could look for other mediating variables to include in the model besides the risk factors associated with the coronavirus pandemic.

## Discussion

The study showed that the presence of risk factors for COVID-19 complication was high at 62.2%; higher than the 21.4% identified in the population of Bangladesh.(19) Presence of risk factors for COVID-19 in these age groups increases and may be due to changes in lifestyles, social or cultural factors, however, people with greater risk in this study have greater fear and concern regarding COVID-19, but no coherence exists with their self-care, given that they take less care of themselves.

Additionally, mental health aspects indicated that fear of COVID-19 obtained a mean of 16.73, lower than that reported in Turkey with a mean of 19.07(20) and New Zealand with a mean of 18.3(21), with those > 60 years of age having the highest score. According with Odzin and Bayrak, fear is directly associated with its transmission measure and rate, as well as with its morbidity and mortality; likewise, high levels of fear make people create mental barriers that avoid rational reaction to COVID-19.(20,21)

With respect to worry about COVID-19, the sample obtained a mean of 11.69, greater than that reported in Turkey with a mean of 2.82 and 2.04 for Peru.(20,22) The foregoing can be explained because it may be associated with diminished wellbeing and a negative perception of overall health, perceiving higher risk of contagion.(22) Nevertheless, fear and worry may increase by uncertainty and insecurity, linked with the perception of severity of the disease, difficulties in its care, conditions under which the response is organized in each place, and risks derived from its exposure.(23) The result reported on self-care for COVID-19 was similar to that reported in Peru by Ruiz *et al.,* in 2020.(24) This sample indicated the lack of a coherent element in the preventive treatment against this disease, given that this perspective helps individuals to gain awareness of their own condition, through improved information and skills to perform adequate self-care actions.(24)

Moreover, our study found that with greater fear there is greater worry about COVID-19, this may be associated with concern having normal and adaptative functions upon situations that threaten or place the person’s life at risk, possibly causing mental health condition.(25,26) It was found that individuals in elderly stage have greater fear, worry, and self-care regarding COVID-19; this may be because of the perception of vulnerability of having complications due to SARS-COV2 with respect to age and presence of comorbidities.(27) This research also found that lower fear means greater self-care; these results were similar to that reported by Hossain *et al.*, the aforementioned allows conducting recommended habits for COVID-19 prevention in the subjects.(19) Thus, individuals with fear of COVID‐19 infection have recurrent controls of bodily temperature and hand washing, that is, they opt for greater self-care.(28) 

Regarding the model, direct effect was found of COVID-19 risk factors on self-care mediated by worry and fear, coupled to it explaining by 14% the self-care conduct for COVID-19, which is due to the elevated presence of risk factors for COVID-19 complication and awareness of fear and concerns to avoid the disease could have caused changes in health behavior, the threat and vulnerability perceived that trigger the adoption of preventive efforts and self-care.(24-29) 

Sample size was found among the study limitations, given that the results cannot be generalized; it is also suggested to conduct further studies in other parts of Mexico and the world, using the instruments indicated, to have a more global vision about fear, worry, and self-care regarding COVID-19.

## Conclusion

Adults in pre-elderly and elderly stages had greater fear, concern, and self-care regarding the disease, with a relation existing between the last two. Coupled with the foregoing, direct effect was produced of the variables number of risk factors for complicated COVID-19, besides fear and concern about the self-care variable, explaining by 14% the self-care conducts for COVID-19. These results are of importance, given their application to improve knowledge on care and on the application of the nursing practice on people in pre-elderly and elderly stages. It is recommended to approach other emotional variables to consider if these increase predictions. Likewise, it is necessary to address the need to diminish or control these risk factors in the population studied to avoid an increase of complications and deaths related to this disease in Mexico.
